# Impact of response to neoadjuvant chemotherapy on surgical modality in patients with T1-2N0-1M0 triple-negative breast cancer

**DOI:** 10.1007/s00432-024-05907-y

**Published:** 2024-08-01

**Authors:** Lidan Chang, Dandan Liu, Qian Hao, Xueting Ren, Peinan Liu, Xingyu Liu, Yumeng Wei, Shuai Lin, Xiaobin Ma, Hao Wu, Huafeng Kang, Meng Wang

**Affiliations:** 1 The Comprehensive Breast Care Center, The Second Affiliated Hospital of Xi’an Jiaotong University, Xi’an, 710061 Shaanxi China; 2 School of Basic Medical Sciences, Xi’an Key Laboratory of Immune Related Diseases, Xi’an Jiaotong University, Xi’an, Shaanxi China

**Keywords:** Neoadjuvant chemotherapy, Triple-negative breast cancer, SEER, Surgical modality

## Abstract

**Purpose:**

Many T1-2N0-1M0 triple-negative breast cancer (TNBC) patients who undergo neoadjuvant chemotherapy (NAC) do not receive breast-conserving therapy (BCT) due to concerns about non-pCR or lymph node metastasis presence.

**Methods:**

T1-2N0-1M0 TNBC patients who underwent NAC between 2010 and 2017 were collected from the SEER database. Factors affecting surgical modalities were analyzed by multinomial logistic regression. The overall survival (OS) and breast cancer-specific survival (BCSS) were evaluated by Kaplan-Meier curves and Cox proportional hazards models. Further stratified subgroup analyses were performed based on the response to NAC and N-stage. Adjusted-hazard ratios were also calculated to exclude potential bias.

**Results:**

A total of 1112 patients were enrolled (median follow-up: 81 months), 58.5% received BCT, 23.6% received reconstruction and 17.9% received mastectomy. Response to NAC and N-stage not only influenced the choice of surgical modality but also were independent predictors for OS and BCSS. The surgery-induced survival differences mainly affect OS. Survival analyses demonstrated that the 10-year OS of BCT was superior or equal to that of mastectomy even in patients with partial response (PR) (77.4% vs. 64.1%, *P* = 0.013), no response (NR) (44.9% vs. 64.2%, *P* = 0.33), or N1 stage (75.7% vs. 57.4%, *P* = 0.0021). In the N1-PR cohort, mastectomy may lead to worse OS (*P* = 0.0012). Besides, between reconstruction and BCT, there was no statistical difference in OS or BCSS (*P* > 0.05).

**Conclusion:**

Our study reveals the necessity of breast surgical de-escalation. Besides, physicians should actively recommend reconstruction for individuals who strongly desire mastectomy.

## Introduction

Breast cancer (BC) is the second leading cause of cancer-related deaths worldwide and the most prevalent cancer in women (Siegel et al. 2024), with triple-negative cases accounting for approximately 15–20% of overall cases (Garrido-Castro et al. 2019). Triple-negative breast cancer (TNBC) is characterized by negative expression of estrogen receptor, progesterone receptor, and human epidermal growth factor receptor 2, and is the most malignant subtype of BC (Wolff et al. 2013). Compared with other molecular subtypes, TNBC is closely associated with an increased risk of recurrence within the first three years, and increased mortality within the first five years (Qiu et al. 2016). Due to the lack of corresponding receptor expression, TNBC patients are not sensitive to endocrine therapy or targeted therapy (Yin et al. 2020). Therefore, the standard systemic treatment for TNBC remains chemotherapy combined with surgery, but conventional postoperative chemotherapy tends to be ineffective, and residual disease (RD) eventually leads to recurrence (Chaudhary et al. 2018; Golshan et al., 2015). Fortunately, TNBC patients are sensitive to neoadjuvant chemotherapy (NAC), and approximately 35-45% of patients achieve pathological complete response (pCR) (Boughey et al. 2014; Mamtani et al. 2016). NAC, which is defined as systemic chemotherapy prior to surgery, was initially used in inoperable advanced BC patients with the aim of downstaging to gain access to surgery. It was gradually applied to patients with early-stage BC to reduce the surgical extent and achieve breast conservation (Cance et al., 2002; Chen et al. 2004). pCR plays an important role in the evaluation of NAC efficacy, has been widely recognized to be associated with long-term survival benefits, and guides surgical approaches as well as postoperative adjuvant treatment (Cance et al., 2002; von Minckwitz et al. 2012). Numerous trials have demonstrated the advantages of NAC in patients with early localized TNBC with a tumor size less than 5 cm (T1-2N0-1 M0). Not only can it create the opportunity for breast-conserving surgery (BCS) instead of mastectomy, but a more sensitive NAC response can also help reduce the extent of tumor excision and improve aesthetics. Its safety has also been repeatedly verified (Atzori et al. 2022; Gwark et al. 2023; Litière et al. 2012).

However, mastectomy rates remain high in patients with T1-2N0-1M0 TNBC, and this overtreatment limits the possibility of breast preservation for many women. For example, in a prospective trial (CALGB 40603), researchers focused on TNBC patients who underwent NAC and observed patients’ ultimate surgical modalities and benefits, 42% of patients who were not eligible for BCS before NAC were converted to candidates for BCS after NAC, with postoperative success rates up to 90%, but fewer than half of patients ultimately opted for BCS (Criscitiello et al. 2016; Golshan et al., 2015). In addition, discordant NAC response and BCS ratios have been observed in other trials, such as the addition of platinum successfully increased the pCR rate for TNBC patients but did not significantly change the BCS acceptance rate (Bear et al. 2003; von Minckwitz et al., 2014). In fact, in a meta-analysis of 10 NAC trials, the Early Breast Cancer Trials Collaborative Group (EBCTCG) proposed that patients who underwent BCS after NAC were more prone to local recurrence, although it did not affect overall survival (OS) (“Long-term outcomes for neoadjuvant versus adjuvant chemotherapy in early breast cancer: meta-analysis of individual patient data from ten randomised trials,” 2018). Numerous studies comparing the impact of different surgical modalities on survival after NAC have shown that RD burden, lymph node status, and tumor subtype are significantly associated with recurrence, with RD being the strongest predictor (Bear et al. 2003; Mukhtar et al. 2023). Besides, Criscitiello C et al. reported selection bias at the surgeon and patient level, including difficulty in accurately assessing RD after NAC, confusion about resection extent, the impact of NAC response degree, and patients’ fear of recurrence (Criscitiello et al. 2013, 2016; Hamelinck et al., 2014). Obviously, pCR can only make binary judgments about NAC response, which is too simplistic for some concerns.

Therefore, in this study, we used the American Joint Committee on Cancer (AJCC) response criteria for NAC to meticulously stratify patients. We focused on T1-2N0-1M0 TNBC patients to explore the impact of different NAC responses (complete response, partial response, no response) on surgical modalities (BCS plus postoperative radiotherapy, mastectomy, reconstruction) and postoperative survival outcomes (overall survival, breast cancer-specific survival). This study aims to integrate subjective and objective factors to provide ideas for delicate individualized treatment.

## Method

### Acquisition of data

**Fig. 1:**
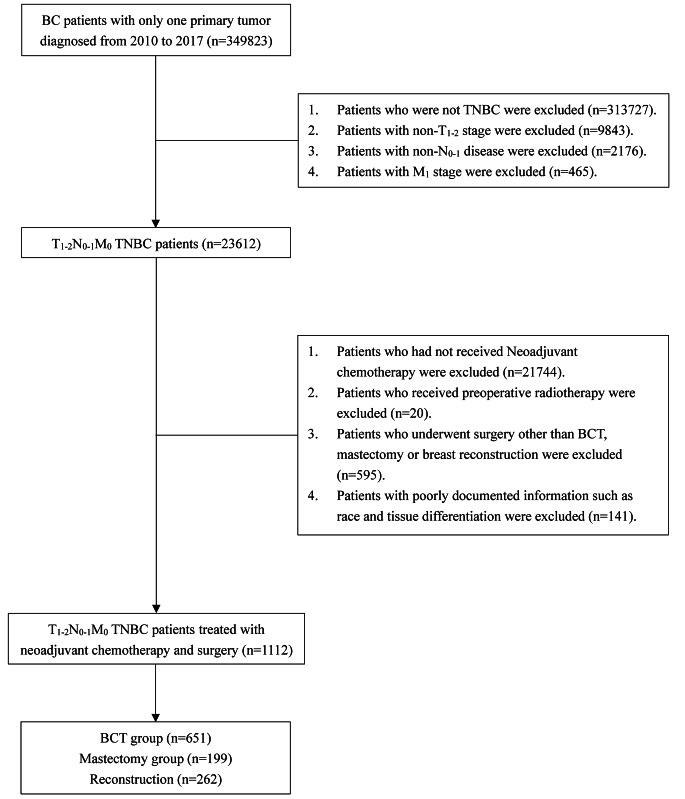
Flowchart of patient selection

Data for this study were obtained from the Surveillance, Epidemiology, and End Results (SEER) database (17regs, 2022nov sub). Patients with T1-2N0-1M0 TNBC who received NAC and surgery between 2010 and 2017 were included in this study. The inclusion criteria for patients were as follows: (1) had breast cancer diagnosed by pathology from 2010 to 2017; (2) had a single primary tumor. The exclusion criteria were as follows: (1) non-TNBC; (2) non-pathologic T1-2N0-1M0 (pT1-2N0-1M0) stage; (3) did not underwent NAC; (4) did not receive BCS and postoperative radiotherapy (BCT), reconstruction, or mastectomy surgery after NAC; (5) received preoperative radiotherapy; and (6) had uncertain or unknown information, such as tissue differentiation degree or ethnicity. Finally, the patients were divided into three groups: the BCT group (who received BCS and postoperative radiotherapy), the mastectomy group (who received mastectomy-only, no reconstruction), and the reconstruction group (who received mastectomy and breast reconstruction). The specific screening process is shown in Fig. 1. The extracted clinicopathological data included the year of diagnosis, age, race, marital status, histological type, differentiation degree, T-N-M stage, surgery, radiotherapy, chemotherapy, response to NAC, and survival data.

### Ethical information

Patient information registered in the SEER database has been de-identified and has an open access policy. Therefore, no additional ethical approval or informed consent is needed.

### Statistical analysis

The patients were divided into three groups according to the surgical modality. The baseline characteristics of the patients are described as frequencies and percentages. Since age is a continuous variable, we used X-tile software to determine the best cut-off value for subsequent group analysis. The analysis endpoints in this study were OS and breast cancer-specific survival (BCSS). Since the significance of different surgical modalities for NAC-received patients remains ambiguous, multinomial logistic regression was employed to identify independent factors affecting surgical options. Next, univariate and multivariate Cox regression analyses were sequentially performed to evaluate the independent prognostic factors of patients. Ultimately, independent factors affecting both surgical modality options and prognosis were identified, based on which further subgroup analysis and stratified analysis were performed. Kaplan-Meier (KM) survival curves and log-rank tests were used to compare the survival of patients in different surgical groups. Adjusted hazard ratios (AHRs) were calculated based on multivariate Cox regression analysis to eliminate potential bias. A two-sided *P* < 0.05 was considered to indicate statistical significance. All the statistical analyses were performed with R software (4.2.3) and associated R packages.

## Results

### Baseline characteristics

**Fig. 2:**
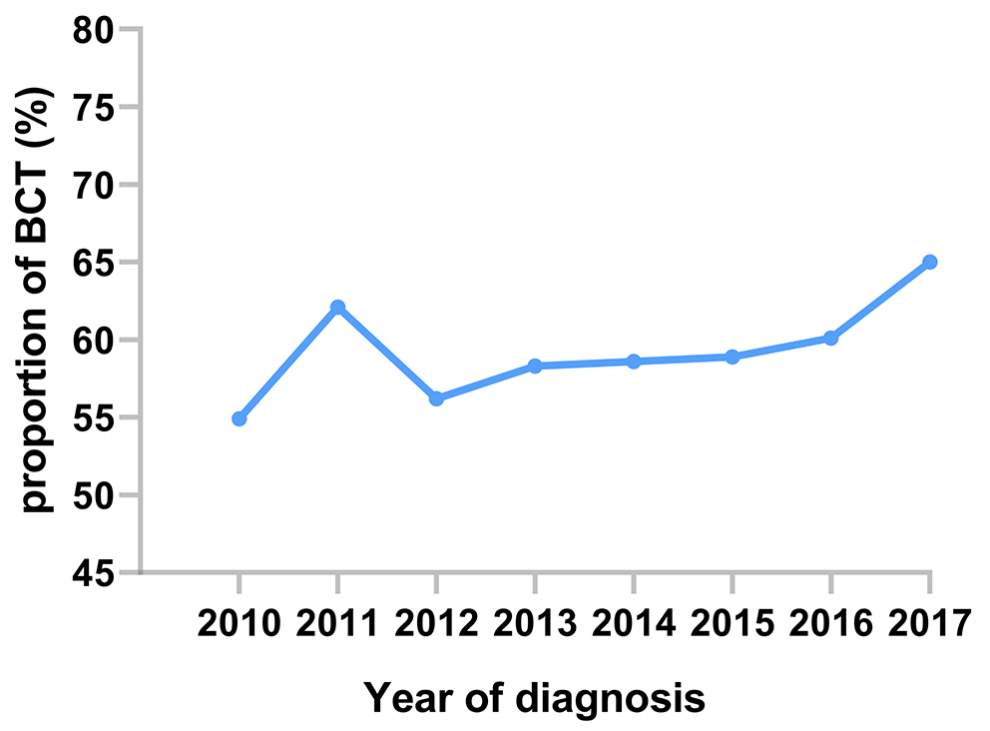
Proportion of patients received BCT from 2010–2017

**Fig. 3:**
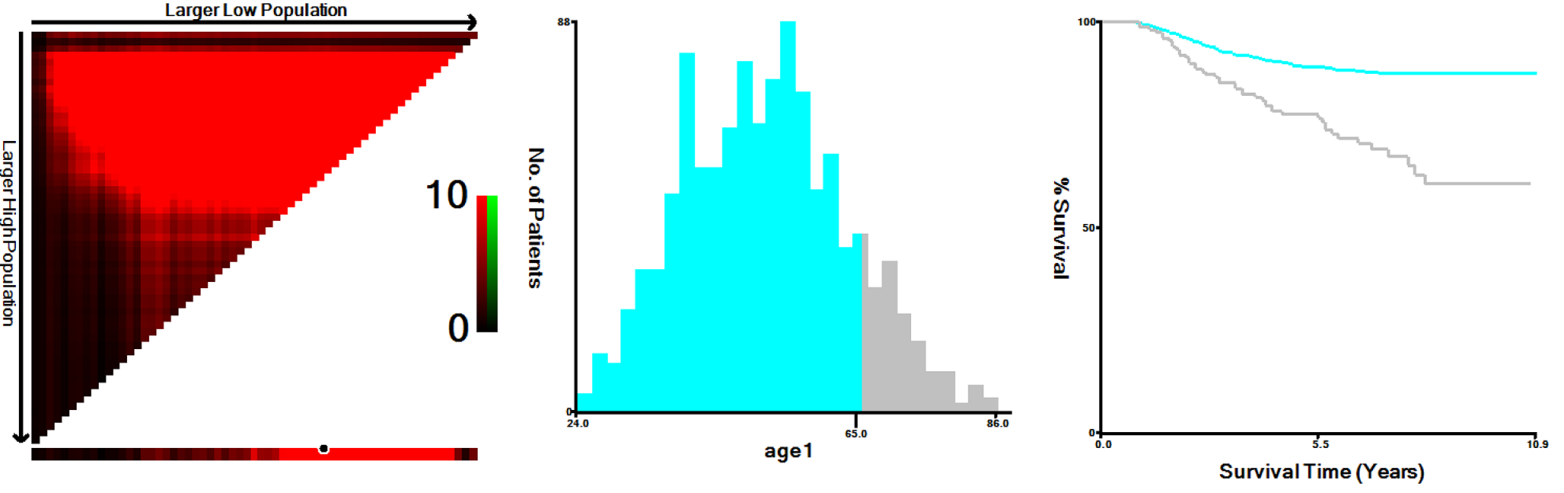
Age of patients divided by X-tile software at the best cut point

From 2010 to 2017, a total of 1112 patients with T1-2N0-1M0 TNBC who underwent surgery after NAC were recruited according to the inclusion-exclusion criteria. The median follow-up time was 81 months (95% CI, 79–84 months). Among them, 651 patients opted for BCT (58.5%), 262 patients opted for reconstruction (23.6%), and 199 patients opted for mastectomy (17.9%). Over the years, the proportion of BCT has shown a slight upward trend, but the overall range has been modest, fluctuating between 55% and 65% (Fig. 2). Based on the KM method, the X-tile program identified an optimal age threshold of 65 years (Fig. 3). Table 1 summarizes the characteristic distributions of demographic, clinicopathologic, and therapeutic information. We could observe that the majority of patients in the three surgical groups were younger than 65 years (BCT: 84.5%; reconstruction: 95.8%; mastectomy: 80.4%), white (69.7%; 79.4%; 74.4%), married status (62.2%; 66.8%; 57.3%), ductal carcinoma ( 91.2%; 89.7%; 93.0%), Grade III or IV (83.6%; 86.3%; 84.9%), T2 stage (72.0%; 67.9%; 72.9%), N0 stage (63.0%; 78.6%; 63.3%), and achieved CR in response to NAC (57.8%; 71.0%; 49.2%). In addition, we observed that in the reconstruction group, a greater percentage of patients were younger, N0 stage, and achieved CR. Whereas, in the BCT or mastectomy group, the proportion of older, N1 stage, PR, or NR patients was relatively higher.

**Table 1:** Baseline demographic characteristics of NAC^1^-received T1-2N0-1M0 TNBC^2^ patients (*n* = 1112)

	BCT^3^ *N* = 651 (%)	Reconstruction *N* = 262 (%)	Mastectomy *N* = 199 (%)	*P*
Age				< 0.001
<=65	550 (84.5)	251 (95.8)	160 (80.4)	
> 65	101 (15.5)	11 ( 4.2)	39 (19.6)	
Race				0.002
Black	149 (22.9)	30 (11.5)	33 (16.6)	
Other^4^	48 ( 7.4)	24 ( 9.2)	18 ( 9.0)	
White	454 (69.7)	208 (79.4)	148 (74.4)	
Marital status				0.112
Married	405 (62.2)	175 (66.8)	114 (57.3)	
Not-married^5^	246 (37.8)	87 (33.2)	85 (42.7)	
Histologic type				0.470
Ductal	594 (91.2)	235 (89.7)	185 (93.0)	
Lobular or other	57 ( 8.8)	27 (10.3)	14 ( 7.0)	
Grade				0.584
Grade I or II	107 (16.4)	36 (13.7)	30 (15.1)	
Grade III or IV	544 (83.6)	226 (86.3)	169 (84.9)	
T				0.396
T1	182 (28.0)	84 (32.1)	54 (27.1)	
T2	469 (72.0)	178 (67.9)	145 (72.9)	
*N*				< 0.001
N0	410 (63.0)	206 (78.6)	126 (63.3)	
N1	241 (37.0)	56 (21.4)	73 (36.7)	
Response to NAC^1^				< 0.001
CR^6^	376 (57.8)	186 (71.0)	98 (49.2)	
PR^7^	238 (36.6)	66 (25.2)	83 (41.7)	
NR^8^	37 ( 5.7)	10 ( 3.8)	18 ( 9.0)	

NAC^1^: neoadjuvant chemotherapy

TNBC^2^: triple-negative breast cancer

BCT^3^: breast-conserving surgery + radiation

Other^4^: American Indian/Alaska Native, Asian/Pacific Islander

Not-married^5^: single (never married), separated, divorced, widowed, unmarried or domestic partner

CR^6^: complete response

PR^7^: partial response

NR^8^: no response

### Survival analysis of the overall study population

**Fig. 4:**
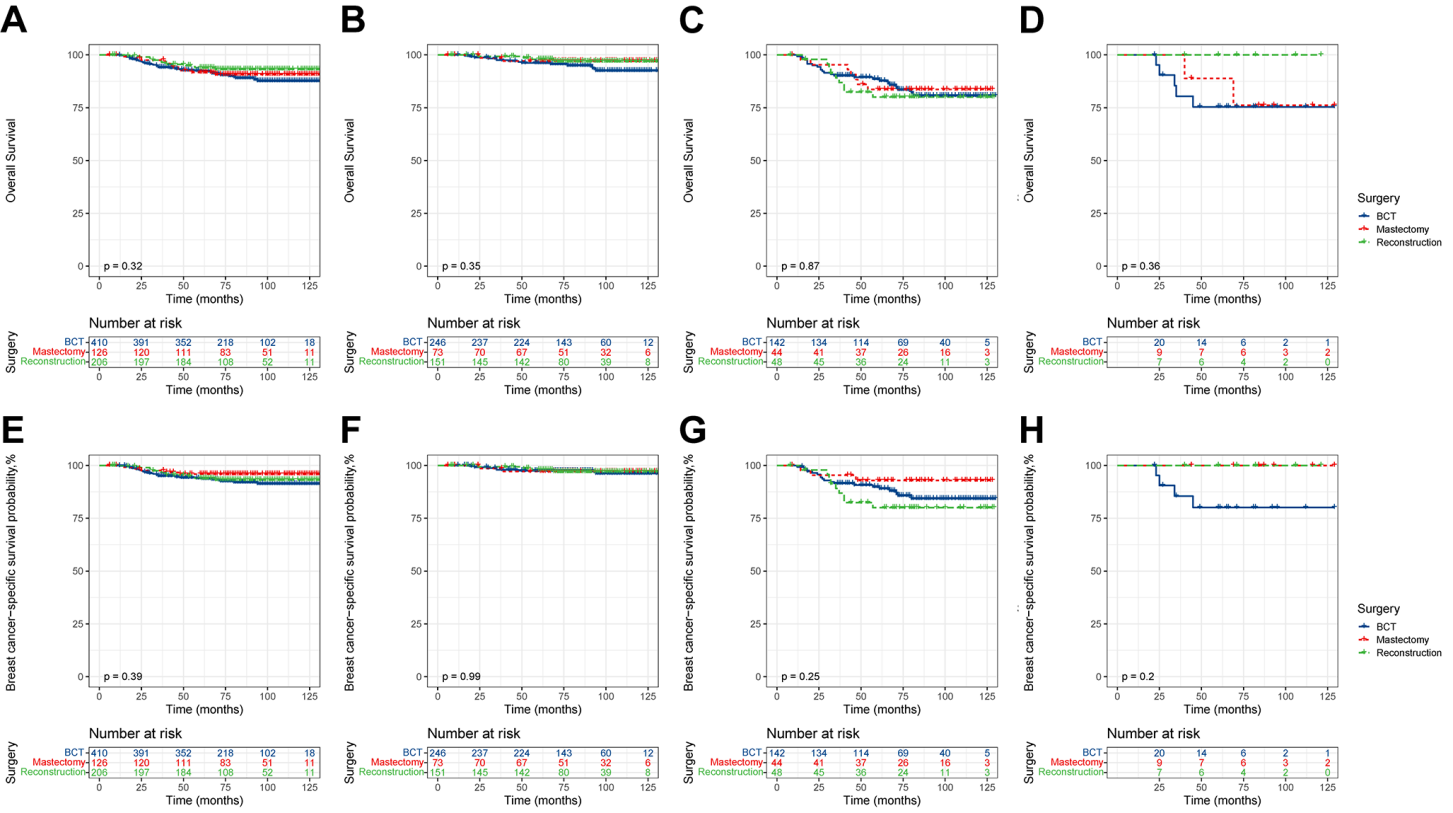
KM survival curves of OS and BCSS in the total population. (**A**), OS of the total population; (**B**), OS of total-CR cohort; (**C**), OS of total-PR cohort; (**D**), OS of total-NR cohort; (**E**), BCSS of the total population; (**F**), BCSS of total-CR cohort; (**G**), BCSS of total-PR cohort; (**H**), BCSS of total-NR cohort

Figure 4 demonstrates the effect of different surgical modalities on survival in the total population. Moreover, further stratification was explored according to the degree of NAC response. KM survival analysis revealed significant intergroup differences in OS between the total population and total-PR cohort (total population: *P* = 0.0042; total-PR cohort: *P* = 0.026). In the total population, patients in the reconstruction group exhibited significantly better OS than those in the BCT and mastectomy groups (reconstruction vs. BCT: HR, 0.59; 95% CI, 0.37–0.93; *P* = 0.022), whereas no significant difference in OS was observed between the BCT and mastectomy groups (mastectomy vs. BCT: HR, 1.39; 95% CI, 0.96–2.01; *P* = 0.081) (Fig. 4A). In the total-PR cohort, the difference in OS between the reconstruction and BCT groups was not statistically significant, but it is noteworthy that both were superior to the mastectomy group (reconstruction vs. BCT: HR, 0.90; 95% CI, 0.48–1.70; *P* = 0.75); (mastectomy vs. BCT: HR, 1.79; 95% CI, 1.13–2.85; *P* = 0.013) (Fig. 4C).

### Identification of independent prognostic factors associated with surgical modalities

We performed a multinomial logistic regression analysis to identify variables affecting surgical modality preference. The results showed that age > 65 years, white or other races, N1 stage, and PR or NR to NAC were independently associated with surgical modality choice (*P* < 0.05) (Table 2). Subsequently, the results of univariate and multivariate Cox regression analyses revealed that N stage and response to NAC were independent prognostic factors in both the OS and BCSS models. Compared with CR, PR and NR were independent risk factors (*P* < 0.001). The N1 stage was another significant independent risk factor compared with the N0 stage (*P* < 0.001). Moreover, in the OS model, patients older than 65 years had worse survival outcomes than relatively younger patients (*P* < 0.001). In terms of the surgical modality, reconstruction appeared to confer a survival advantage compared to BCT (*P* = 0.023). However, this impact was not significant in the BCSS model (Table 3).

**Table 2:** Multinomial logistic regression of factors associated with surgical modalities among NAC^1^-received T1-2N0-1M0 TNBC^2^

	Reconstruction vs. BCT^3^		Mastectomy vs. BCT^3^	
	OR(95%CI)	*P*	OR(95%CI)	*P*
(Intercept)	0.34(0.18–0.62)	< 0.001	0.14(0.07–0.27)	< 0.001
Age				
<=65	Reference		Reference	
> 65	0.24(0.13–0.46)	< 0.001	1.26(0.83–1.92)	0.272
Race				
Black	Reference		Reference	
Other^4^	2.38(1.24–4.57)	0.009	1.81(0.92–3.57)	0.086
White	2.28(1.46–3.54)	< 0.001	1.56(1.01–2.41)	0.045
Marital_status				
Married	Reference		Reference	
Not-married^5^	0.94(0.68–1.29)	0.697	1.34(0.96–1.87)	0.088
Histologic type				
Ductal	Reference		Reference	
Lobular or other	1.3(0.79–2.15)	0.306	0.79(0.43–1.47)	0.459
Grade				
Grade I or II	Reference		Reference	
Grade III or IV	1.17(0.77–1.80)	0.465	1.15(0.74–1.81)	0.533
T				
T1	Reference		Reference	
T2	0.8(0.58–1.11)	0.188	1.03(0.72–1.49)	0.854
*N*				
N0	Reference		Reference	
N1	0.51(0.36–0.72)	< 0.001	0.96(0.69–1.35)	0.822
Response to NAC^1^				
CR^6^	Reference		Reference	
PR^7^	0.62(0.44–0.87)	0.006	1.32(0.94–1.86)	0.113
NR^8^	0.61(0.29–1.28)	0.189	1.89(1.02–3.48)	0.042

NAC^1^: neoadjuvant chemotherapy

TNBC^2^: triple-negative breast cancer

BCT^3^: breast-conserving surgery + radiation

Other^4^: American Indian/Alaska Native, Asian/Pacific Islander

Not-married^5^: single (never married), separated, divorced, widowed, unmarried or domestic partner

CR^6^: complete response

PR^7^: partial response

NR^8^: no response

**Table 3:** OS and BCSS in univariate and multivariate analyses

	OS				BCSS			
	Univariate		Multivariate		Univariate		Multivariate	
	HR (95%CI)	*P*	HR (95%CI)	*P*	HR (95%CI)	*P*	HR (95%CI)	*P*
Age								
<=65	Reference		Reference		Reference		Reference	
> 65	2.83(2.00–4.00)	< .001	2.18(1.52–3.14)	< .001	1.53(0.96–2.43)	0.071	1.21(0.75–1.95)	0.443
Race								
Black	Reference		Reference		Reference		Reference	
Other^1^	1.07(0.56–2.05)	0.845	1.28(0.65–2.51)	0.473	1.17(0.57–2.41)	0.661	1.29(0.61–2.70)	0.505
White	1.02(0.68–1.52)	0.941	1.11(0.73–1.69)	0.612	1.02(0.65–1.62)	0.923	1.12(0.70–1.80)	0.646
Marital status								
Married	Reference		Reference		Reference		Reference	
Not-married^2^	1.18(0.86–1.62)	0.302	1.22(0.88–1.70)	0.234	1.16(0.81–1.67)	0.412	1.24(0.85–1.80)	0.262
Histologic type								
Ductal	Reference		Reference		Reference		Reference	
Lobular or other	1.21(0.72–2.02)	0.479	1.21(0.71–2.04)	0.481	1.15(0.63–2.09)	0.641	1.20(0.66–2.20)	0.550
Grade								
Grade I or II	Reference		Reference		Reference		Reference	
Grade III or IV	0.91(0.60–1.39)	0.658	1.11(0.72–1.71)	0.623	0.92(0.57–1.49)	0.744	1.08(0.66–1.76)	0.769
T								
T1	Reference		Reference		Reference		Reference	
T2	0.93(0.66–1.31)	0.672	0.88(0.62–1.25)	0.47	1.08(0.72–1.60)	0.720	1.00(0.67–1.50)	0.998
*N*								
N0	Reference		Reference		Reference		Reference	
N1	3.09(2.25–4.24)	< .001	2.77(2.00-3.82)	< .001	3.35(2.33–4.81)	< .001	3.01(2.08–4.35)	< .001
Response to NAC^3^								
CR^4^	Reference		Reference		Reference		Reference	
PR^5^	3.83(2.67–5.50)	< .001	3.16(2.18–4.58)	< .001	3.92(2.59–5.93)	< .001	3.48(2.28–5.32)	< .001
NR^6^	6.97(4.23–11.46)	< .001	5.67(3.40–9.47)	< .001	7.61(4.36–13.26)	< .001	7.08(4.01–12.52)	< .001
Surgery								
BCT^7^	Reference		Reference		Reference		Reference	
Mastectomy	1.39(0.96–2.01)	0.085	1.08(0.73–1.58)	0.712	1.11(0.71–1.75)	0.639	0.92(0.57–1.46)	0.714
Reconstruction	0.59(0.37–0.93)	0.023	0.88(0.55–1.40)	0.595	0.67(0.42–1.09)	0.111	0.96(0.59–1.58)	0.887

Other^1^: American Indian/Alaska Native, Asian/Pacific Islander

Not-married^2^: single (never married), separated, divorced, widowed, unmarried or domestic partner

NAC^3^: neoadjuvant chemotherapy

CR^4^: complete response

PR^5^: partial response

NR^6^: no response

BCT^7^: breast-conserving surgery + radiation

### Subgroup analysis according to N stage

**Fig. 5:**
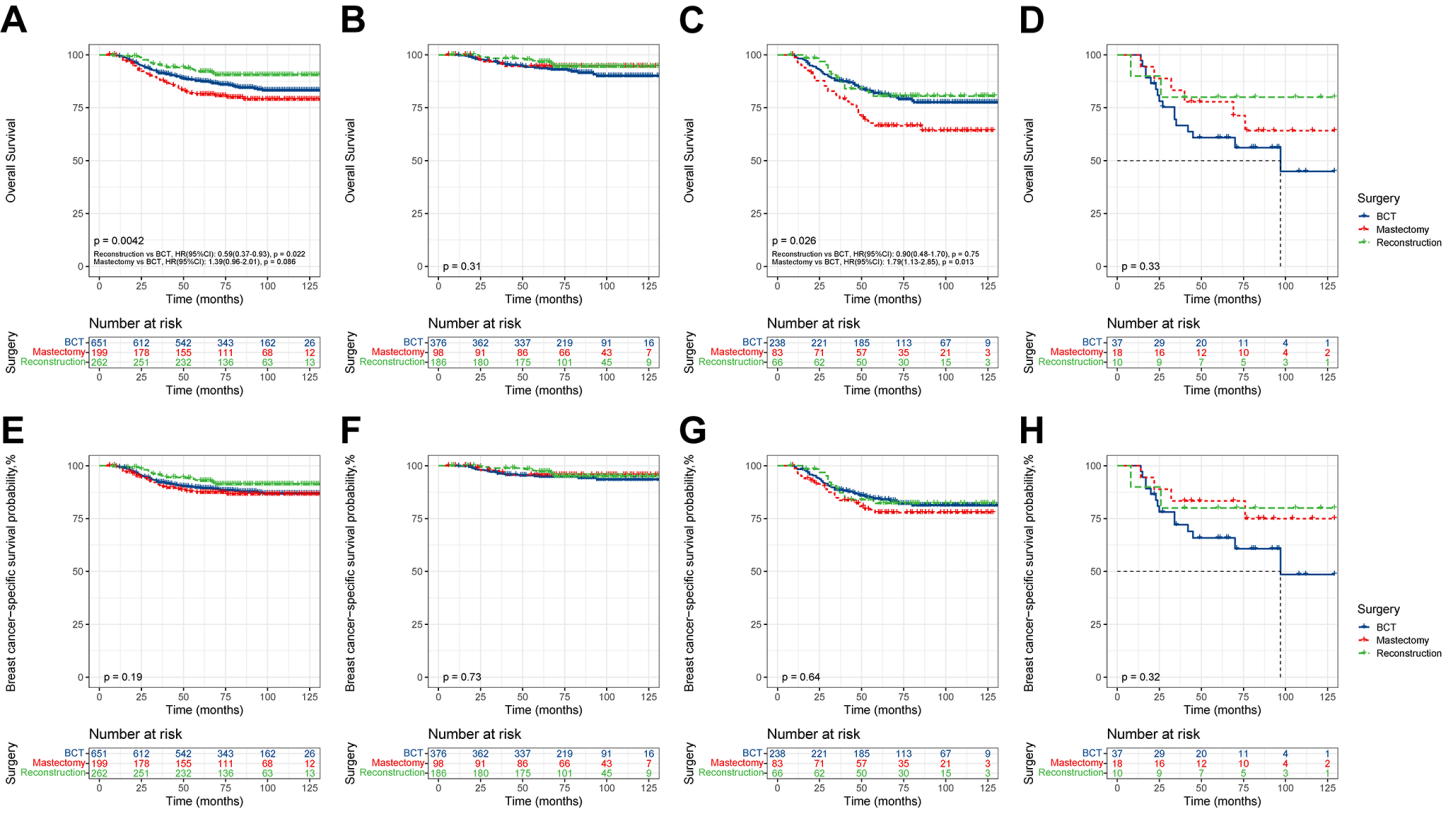
KM survival curves of OS and BCSS N0 subgroup. (**A**), OS of the total N0 population; (**B**), OS of N0-CR cohort; (**C**), OS of N0-PR cohort; (**D**), OS of N0-NR cohort; (**E**), BCSS of the total N0 population; (**F**), BCSS of N0-CR cohort; (**G**), BCSS of N0-PR cohort; (**H**), BCSS of N0-NR cohort

**Fig. 6:**
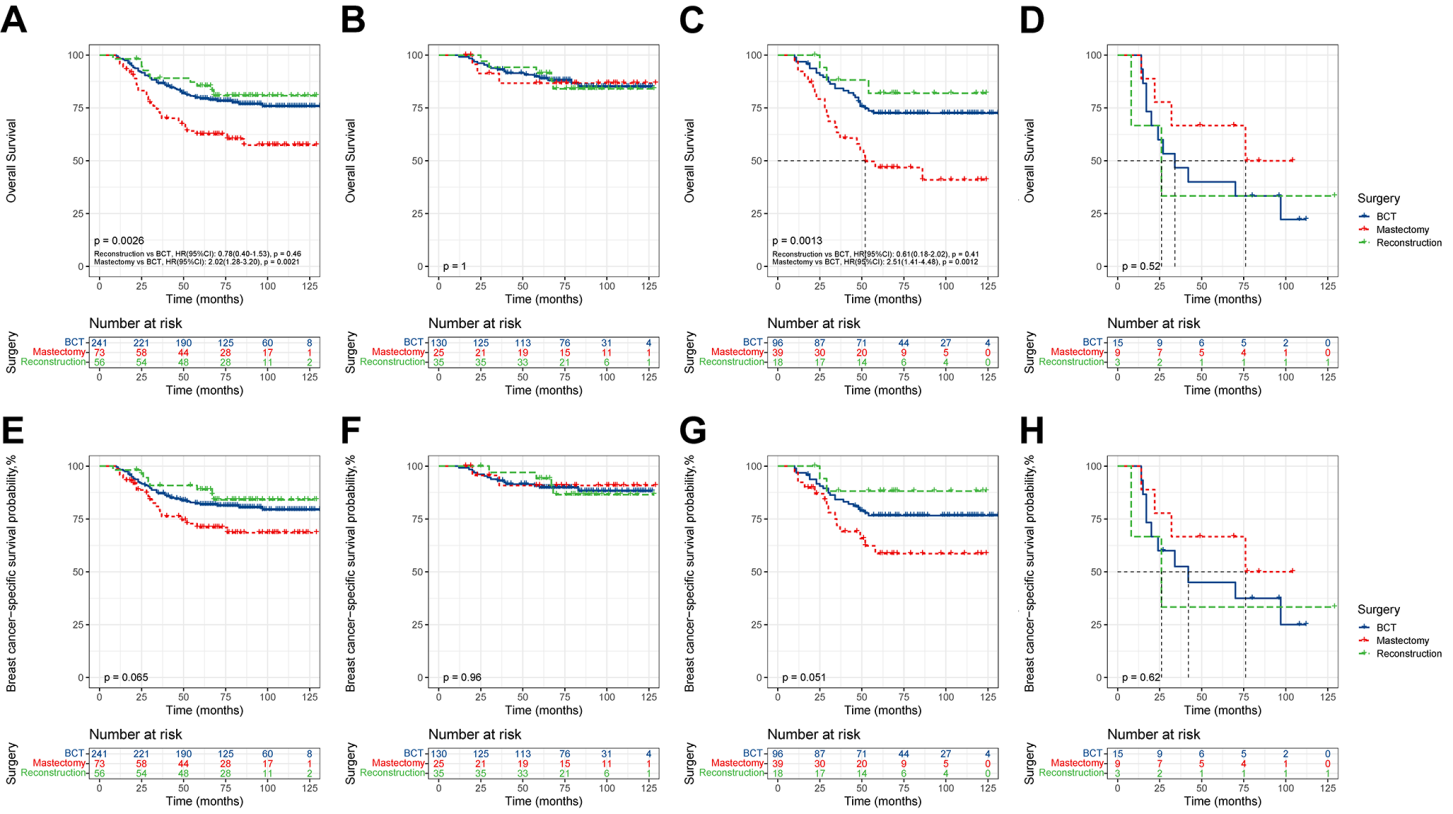
KM survival curves of OS and BCSS N1 subgroup. (**A**), OS of the total N1 population; (**B**), OS of N1-CR cohort; (**C**), OS of N1-PR cohort; (**D**), OS of N1-NR cohort; (**E**), BCSS of the total N1 population; (**F**), BCSS of N1-CR cohort; (**G**), BCSS of N1-PR cohort; (**H**), BCSS of N1-NR cohort

The population was divided into N0 and N1 subgroups, and meticulously stratified according to the response to NAC in each subgroup. For the N0 subgroup, there was no significant difference in OS or BCSS among the three surgical modalities in either the total population or the stratified cohorts, suggesting that patients without lymph node metastasis have a favorable survival prognosis (Fig. 5). Regarding to the N1 subgroup, KM analysis revealed significant intergroup differences in survival outcomes among the three surgeries (*P* = 0.0026). Specifically, both the BCT and reconstruction groups exhibited better OS than did the mastectomy group, with no significant difference between the two groups (reconstruction vs. BCT: HR, 0.78; 95% CI, 0.40–1.53; *P* = 0.46; mastectomy vs. BCT: HR, 2.02; 95% CI, 1.28–3.20; *P* = 0.0021) (Fig. 6A). Remarkably, this surgery-induced survival difference was extraordinarily significant in the N1-PR cohort (*P* = 0.0013, Fig. 6C). However, for the N1-CR cohort, the three surgeries did not demonstrate significant intergroup differences, and all three surgeries were associated with positive survival outcomes (Fig. 6B and F). In contrast, for the N1-NR cohort, although the mastectomy group exhibited a relatively high survival rate, the difference in surgical modalities was not statistically significant, which may be attributed to the limited sample size (Fig. 6D and H).

To address the potential bias in patients with different lymph node metastases, further calculations of AHR for different surgical modalities were conducted (Table 4). For patients in the N1-CR or N1-NR cohorts, there was no additional survival benefit from mastectomy or reconstruction compared with BCT. For the N1-PR cohort, the AHR for OS among patients who underwent mastectomy compared with BCT was 2.19 (95%CI, 1.19–4.06; *P* = 0.012). The results of the above subgroup analyses revealed the role of NAC response in surgical decision-making.

**Table 4:** NAC^1^ response-adjusted hazard ratios of OS and BCSS

			Mastectomy vs BCT^2^		Reconstruction vs BCT^2^	
			AHR (95% CI)	*P*	AHR (95% CI)	*P*
N0 stage	Response to NAC^1^	CR^3^				
		OS	0.68(0.15–3.10)	0.613	0.84(0.25–2.83)	0.781
		BCSS	1.13(0.22–5.70)	0.881	1.37(0.36–5.18)	0.645
		NR^4^				
		OS	0.46(0.08–2.64)	0.385	NA^6^	NA^6^
		BCSS	NA^6^	NA^6^	NA^6^	NA^6^
		PR^5^				
		OS	0.90(0.38–2.10)	0.799	1.27(0.58–2.76)	0.548
		BCSS	0.90(0.38–2.10)	0.799	1.27(0.58–2.76)	0.548
N1 stage	Response to NAC^1^	CR^3^				
		OS	0.94(0.26–3.45)	0.929	1.12(0.39–3.21)	0.826
		BCSS	0.78(0.17–3.55)	0.746	0.91(0.29–2.87)	0.868
		NR^4^				
		OS	0.53(0.10–2.78)	0.456	0.36(0.02–5.42)	0.460
		BCSS	0.71(0.14–3.63)	0.677	0.51(0.03–7.91)	0.628
		PR^5^				
		OS	2.19(1.19–4.06)	0.012	0.63(0.19–2.10)	0.451
		BCSS	1.78(0.87–3.62)	0.112	0.44(0.10–1.92)	0.278

NAC^1^: neoadjuvant chemotherapy

BCT^2^: breast-conserving surgery + radiation

CR^3^: complete response

NR^4^: no response

PR^5^: partial response

NA^6^: not applicable

## Discussion

In this large retrospective study, we first confirmed the efficacy of using the AJCC response criteria for NAC to guide surgical modality selection and predict patient prognosis. Second, we confirmed the safety of BCT through stratified analyses. Even in patients who did not achieve CR or were N1 stage, BCT was superior or equal to mastectomy, regardless of OS or BCSS. AHR results showed that in the N1-PR cohort, patients who underwent mastectomy showed a 119% higher risk of death compared with BCT, suggesting that mastectomy may lead to worse OS but does not affect BCSS. Besides, our study additionally focused on patients who underwent reconstruction after mastectomy. We found that reconstruction seemed to provide additional benefits when considering OS in the overall study cohort. However, after meticulous stratification and subgroup analyses, the survival difference between reconstruction and BCT was no longer statistically significant. These results reveal the necessity of breast surgery de-escalation, and physicians should recommend BCT rather than more extensive surgery. Second, further reconstruction should be actively recommended for those who prefer mastectomy, not only for aesthetics but also for practical clinical benefit.

Over the past decade, the efficacy of preoperative systemic chemotherapy in operable TNBC patients has been widely recognized. NAC with paclitaxel plus anthracycline as the backbone significantly improved OS (Chaudhary et al. 2018; Harbeck & Gluz, 2017; Murphy et al. 2018). Especially after the CREATE-X trial demonstrated that capecitabine could be used to improve the survival of TNBC patients with RD (Masuda et al. 2017). The preference of NAC for early TNBC is fully accepted. Particularly for localized early-stage patients with T1-2N0-1M0 TNBC, the downsizing effect of NAC not only improves the BCT rate but also reduces the surgical scope (Piltin et al. 2020). For the N0 stage, NAC helps to eliminate occult lesions. For the N1 stage, NAC offers the opportunity to avoid lymph node dissection (Fisher et al., 2020; Steenbruggen et al. 2017). In recent years, researchers have continued to explore the incorporation of additional chemotherapeutic agents (e.g., platinum), poly (ADP-ribose) polymerase (PARP) inhibitors, and immune checkpoint inhibitors into the chemotherapy backbone to improve pCR rates (Litton et al. 2018; Schmid et al. 2018; Tutt et al. 2018). The effect was notable, especially in patients who were programmed cell death ligand 1 (PD-L1)-positive or had germline BRCA mutations (Schmid et al. 2020). However, studies have shown that approximately 35% of patients still do not achieve CR after NAC (Aldrich et al. 2023; Schmid et al. 2020). RD is directly associated with local recurrence, and several studies have documented that failure to CR makes impacts BCT conversion rates (Liedtke et al. 2008). According to the distribution of the baseline population included in this study, 461 of the 1112 patients (41.5%) who underwent NAC still received mastectomy or reconstruction. Of these patients, more than half of those who chose mastectomy did not achieve CR (50.7%), which is consistent with the findings of the above studies, confirming that incomplete response to NAC is an important factor affecting the clinical conversion rate of BCT. The RDs resulting from the incomplete response are worrisome: objectively, they demonstrate relatively resistant tumor biology and significantly correlate with local recurrence; subjectively, they lead to patient anxiety about prognosis (Criscitiello et al. 2016; Masuda et al. 2022; von Minckwitz et al. 2019). The degree of NAC response is involved in clinical decision-making.

Subsequently, the results of multinomial logistic and multivariate Cox regression analyses disclosed that response to NAC, as well as N stage, not only affects surgical choice but is also strongly associated with OS and BCSS. The multinomial logistic model also suggested specific effects on surgical modalities: patients with PR or N1 stage disease preferred BCT over reconstruction [reconstruction vs. BCT: N1, OR (95% CI): 0.51(0.36–0.72), *P* < 0.001; PR, OR (95% CI): 0.62(0.44–0.87), *P* = 0.006], while NR patients preferred mastectomy [mastectomy vs. BCT: NR, OR (95% CI): 1.89(1.02–3.48), *P* = 0.042]. The independent role of lymph node status on BC prognosis is well known. Regardless of the molecular subtype, lymph node metastasis more than doubles the risk of recurrence. The specific number of involved nodes is also strongly associated with survival outcomes (Morrow et al. 2018; Nathanson et al., 2018). Numerous reports have confirmed that in patients with small tumors at the N1 stage, timely NAC can effectively decelerate axillary progression and prevent axillary lymph node dissection (Boughey et al. 2014; Samiei et al. 2021). In our study, we explored the role of N stage in the clinical management of NAC-received patients. Furthermore, we initiated a subgroup analysis based on distinct N stages to investigate the association between response to NAC and surgical modality. We conclude that lymph node status mainly affects surgical selection and prognosis in the N1 subgroup, especially in the N1-PR cohort. In the N1 subgroup, the mastectomy group did not demonstrate the expected survival benefit compared to the BCT group but instead contributed to a poorer OS. Further AHR results also verified that in the N1-PR cohort, the risk of mastectomy was 2.19-fold greater than that of BCT, which became an independent risk factor for OS. This may be due to the existence of resistant biology, and timely postoperative adjuvant therapy such as capecitabine is warranted to reduce the risk of recurrence (Cortes et al. 2020; Patel and DeMichele 2017). Nevertheless, mastectomy is traumatic and requires a long recovery time both physically and psychologically, which may delay intensive adjuvant therapy. Meanwhile, subgroup analysis of the NR cohort showed no survival differences in OS or BCSS among the three surgical groups, further demonstrating the safety of BCT. Although the survival rate for the mastectomy group appears to be slightly higher, this may be due to an insufficient number of patients, which did not result in a statistical difference.

The psychosocial and emotional benefits of reconstruction have been widely documented (Atisha et al., 2008; Heneghan et al., 2011). For patients who are eligible for BCT but are not sensitive to NAC or have lymph node metastases which make them apprehensive about BCT and seek a more complete localized control effect, the physician needs to respect their subjective wishes, at which point reconstruction becomes the ideal option. Our findings suggest that reconstruction is as extensive as mastectomy, but survival outcomes can be comparable to those of BCT. Surprisingly, the reconstruction group appeared to provide an additional OS benefit when looking at the overall population, which requires further exclusion of other effects. Besides, logistic regression results revealed that age and race were also involved in the trade-off between reconstruction and BCT. Patients who were < = 65 years old, white, or other non-black races were more likely to receive reconstruction. This may be because younger patients are more concerned about body image, and the non-black communities have higher socioeconomic levels, better treatment compliance and insurance status (Hershman et al. 2005; Killelea et al. 2015; Tammemagi, 2007). Therefore, their demand for medical services is not only about curing disease, but also for improving quality of life (Reyes et al. 2016). It should be noted that racial differences did not affect the OS or BCSS of the study cohort, whereas the age-induced survival difference was only in the OS model, with those older than 65 years experiencing worse OS. Possibly because surgical injury and chemotherapy toxicity cannot be ignored in older patients.

In conclusion, our study provides direct evidence for physicians to more firmly prefer BCT, even if patients have lymph node metastases or are insensitive to NAC. Alternatively, reconstruction should be actively recommended for patients who intensely demand mastectomy. Certainly, our study has some limitations. First, the inherent bias of retrospective studies is difficult to avoid. Second, we compared the impact of the response to NAC on surgical modality and survival under different high-risk factors, but did not distinguish the specific chemotherapeutic regimen, which was not available from the SEER database. Finally, the SEER database lacks more specific information such as Ki67, genetic testing results, and tumor markers, which may affect surgical modalities and prognosis. We plan to further validate the conclusions in future clinical work.

## Data Availability

No datasets were generated or analysed during the current study.
